# Supplementary material for: Addressing cluster-constant covariates in mixed effects models via likelihood-based boosting techniques
Source: PLoS One. 2021 Jul 9;16(7):e0254178. doi: 10.1371/journal.pone.0254178 (PMC8270154; doi:10.1371/journal.pone.0254178)
Supplement: Attachment — Submitted filename: response.pdf [file pone.0254178.s002.pdf]

Response to reviewers' comments on manuscript PONE-D-21-03209

Colin Griesbach, Andreas Groll, Elisabeth Bergherr

May 7, 2021

The authors would like to thank the two reviewers and the academic editor for the quick and positive feedback. Please find a detailed point-to-point response listed below.

1 Academic Editor

- Please review your reference list to ensure that it is complete and correct. If you have cited papers that have been retracted, please include the rationale for doing so in the manuscript text, or remove these references and replace them with relevant current references. Any changes to the reference list should be mentioned in the rebuttal letter that accompanies your revised manuscript. If you need to cite a retracted article, indicate the articles retracted status in the References list and also include a citation and full reference for the retraction notice.

  Done.

- Please ensure that your manuscript meets PLOS ONE's style requirements, including those for file naming.

  Done. We used the PLOS ONE LaTeX-template and thoroughly followed the instructions.

- Please provide an amended Competing Interests Statement that explicitly states this commercial funder, along with any other relevant declarations relating to employment, consultancy, patents, products in development, marketed products, etc.

  We addressed this funding issue in the coverletter.

2 Reviewer 1

- The topic is interesting and the paper is overall well written. There is no suggestion to this paper, i.e. the authors should consider to make proper

graphs out of major results instead of intense texts with tables, this paper would become much more readable to the potential authors.

Thanks for this comment. We agree, that pure tables can be tiring to read and added two additional Figures. One showcasing the computational effort and one depicting the coefficient progression for the data example which highlights the boosting mechanism in more detail.

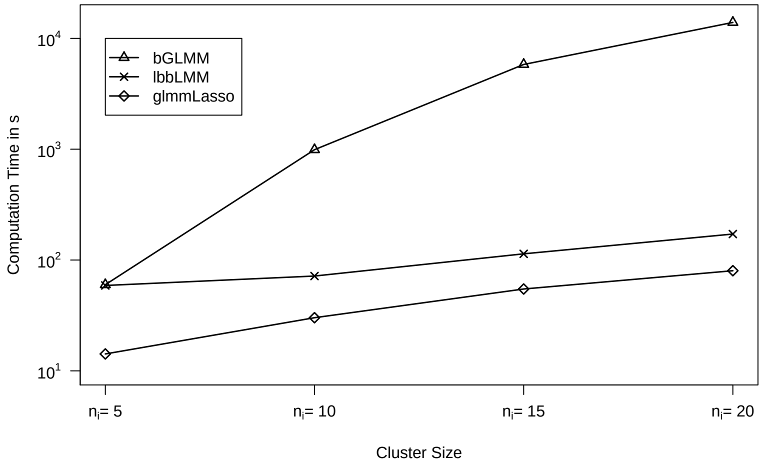

Figure 2: Differing computational effort of the regularization routines `bGLMM`, `lbbLMM` and `glmmLasso` for varying cluster sizes.

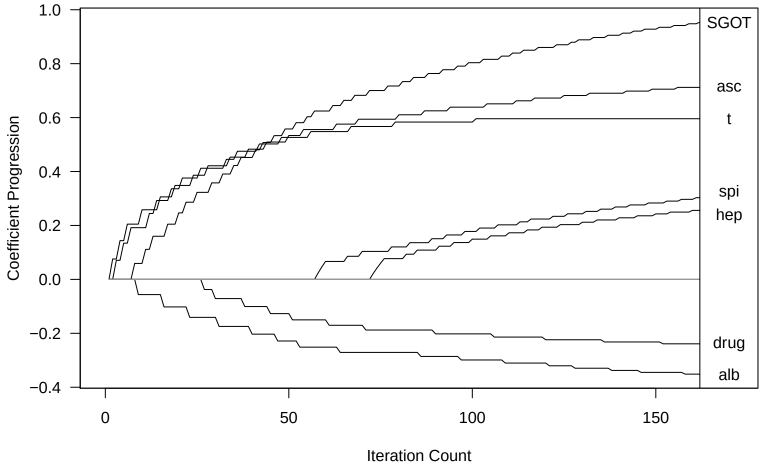

Figure 3: Coefficient progression for the PBC data obtained by `lbbLMM` with $m_* = 161$.

3 Reviewer 2

- This study proposed a novel and refined boosting algorithm for mixed models, particularly in the presence of cluster-constant covariates. The advantages of the method in terms of estimation accuracy and computational time were well demonstrated by an extensive simulation study. The application potential of the method was also further illustrated by an empirical study using the primary biliary cirrhosis data. Overall, the topic is interesting and worthy of investigation. The conclusions are robust and reliable. Since the method is technically sound and the contributions are substantial, I have no further comments and thus suggest it for publication.

  Thank you for the positive feedback.
